# Governments’ economic support for households during the COVID-19 pandemic and consumer confidence

**DOI:** 10.1007/s00181-023-02367-0

**Published:** 2023-02-01

**Authors:** Hassan F. Gholipour, Reza Tajaddini, Mohammad Reza Farzanegan

**Affiliations:** 1 School of Business, Western Sydney University, Sydney, Australia; 2 School of Business, Law, and Entrepreneurship, Swinburne University of Technology, Melbourne, Australia; 3 Economics of the Middle East Research Group, Center for Near and Middle Eastern Studies (CNMS), School of Business and Economics, Philipps-Universität Marburg, Marburg, Germany; 4 CESifo, Munich, Germany; 5 ERF, Cairo, Egypt

**Keywords:** Consumer confidence, COVID-19 pandemic, Debt relief, Economic support, Income support, System GMM, OECD, D12, E62, H31

## Abstract

To combat the adverse consequences of the COVID-19 pandemic, governments have implemented various economic policies. This study examines how different types of government economic support for households are associated with consumer confidence. Utilizing data from 35 countries in the Organization for Economic Co-operation and Development for January 2020–October 2021 and applying panel fixed effect and system generalized methods of moments regressions, we show that higher levels of government economic support lead to higher levels of consumer confidence. The results also suggest that government income support for households has a stronger impact than debt/contract relief on consumer confidence during the pandemic in the full sample. Moreover, we find that debt/contract relief is a more effective policy to boost confidence in emerging economies. Finally, COVID-19 fatalities have a significant negative effect on consumer confidence.

## Introduction

The COVID-19 pandemic has profoundly impacted the economy and human lives around the world. To combat the socio-economic implications of the pandemic, governments have introduced support packages, unprecedented in their scales. This study investigates which government economic policy to support households during the pandemic has been able to restore consumer confidence.

Consumer confidence is a crucial factor explaining households’ aggregate consumption (e.g., Gillitzer and Prasad 2018; Fuhrer 1993; Acemoglu and Scott 1994; Hemming et al. 2002), households’ savings and investment (Vanlaer et al. 2020; Mishkin 1978; Hemming et al. 2002), household financial decisions (Białowolski 2019), economic growth (Matsusaka and Sbordone 1995; Guo and He 2020), productivity (Barsky and Sims 2012), and a variety of political behavior such as trust in government (Chanley et al. 2000) and voters’ support for government (Indridason 2014). Bachman and Sims (2012) argue that household confidence plays a crucial role in the transmission of government spending shocks into economic activity. Biktimirov et al. (2021) show that the sentiment generated by media measured by the breadth and intensity of the COVID-19 news coverage is significantly related to the S&P 500 index returns during 2020.

Given the importance of consumer confidence, several studies have attempted to explore its economic and political determinants using longitudinal data. Since there is no off-the-shelf workhorse model for consumer confidence (Bachman and Sims 2012) empirical studies have examined various economic and political indicators in their consumer confidence modeling. Some of the economic drivers include inflation rate, economic news, unemployment rate, interest rate, gross domestic product (GDP), and industrial production (e.g., De Boef and Kellstedt 2004; Hollanders and Vliegenthart 2011; Ramalho et al. 2011; Paradiso et al. 2014; Konstantinou and Tagkalakis 2011). Researchers have also shown that wars, elections, economic policy, and economic management can be considered among the political determinants of consumer sentiment (De Boef and Kellstedt 2004; Adam 2014).

There is also a strand of literature that mainly focuses on how macroeconomic policies (fiscal and monetary) impact consumers’ perception of the future of the economy (e.g., Alesina et al. 2015; De Boef and Kellstedt 2004; Konstantinou and Tagkalakis 2011). The majority of these studies support Keynes’ (1936) *General Theory of Employment, Interest, and Money* that states fiscal stimuli reduce precautionary saving and lead to higher demand and consumer spending. This is expected to sustain the domestic economy and boost consumer confidence.

For example, Konstantinou and Tagkalakis (2011) show that reductions in personal and business direct taxes have a positive effect on consumer and business sentiment and lead to higher spending. Tax cuts, which are usually perceived as temporary supports, particularly aid those facing binding liquidity constraints. However, the government wage bill and government investment are found to have opposite effects on consumer and business confidence. Konstantinou and Tagkalakis (2011) suggest it is because the latter policies are perceived to result in a larger public sector in future, which needs to be financed by higher taxes. Similarly, Papaioannou (2019) shows that expansionary fiscal policy has a positive and significant impact on confidence in Greece over the period 1999 to 2016. Hemming et al. (2002) argue that households’ or firms’ confidence in the general economic environment can be influenced by government economic policies (e.g., anticipated future deficits hurt confidence).

Contrary to Keynes’s view on fiscal policies, there are economic theories that follow the *Rational Expectations* school of thought. For example, the *Ricardian Equivalence Theory* (Ricardo 1951; Barro 1974) argues that rational consumers boost their savings rather than their spending in response to the government’s quantitative easing and tax-cut policies. It is because these consumers foresee higher taxes in future that are required to cover government budget deficits. This theory assumes that as government spending has to ultimately be financed by the private sector, any benefits from the Keynesian fiscal policy will be canceled out (see Giavazzi and Pagano 1990; Blanchard and Perotti 2002; Alesina and Ardagna 2010; Konstantinou and Tagkalakis 2011; Wijnbergen and Kwaak 2017). This phenomenon is also known as the crowding-out theory and rests on the assumption of a finite supply of money, which means governments limit borrowing and investment opportunities for the private sector which adversely impacts economic growth. However, the existence of a global capital market questions the very notion of a finite money supply. The recent economic stimuli introduced by governments around the world have initiated novel arguments that debt sustainability may not be an issue as long as interest rates remain below the nominal growth rate (Krugman 2020).

This paper extends the literature on the link between macroeconomic policies and sentiment by examining the impact of the government’s economic support for households during the COVID-19 pandemic on consumer confidence across 35 OECD countries from January 2020 to October 2021. The outbreak of COVID-19 rapidly affected more than 200 countries since the official confirmation of the first case in mid-December 2019. It quickly became the most serious health crisis in a century and the most severe economic crisis since the Second World War (OECD 2021a, b, c). To curb the simultaneous health, economic and social challenges caused by the pandemic, governments around the world introduced specific health measures (e.g., limitations on social gatherings, vaccine mandates, face mask-wearing) and economic policy responses (e.g., COVID relief payments, interest rates cuts, quantitative easing) (Gholipour and Arjomandi 2021; Farzanegan and Gholipour 2021).

The fiscal stimuli aimed to boost business and consumer confidence and ultimately improve household consumption and business investments (OECD 2020; IMF 2021). The extent of fiscal and monetary policies during the pandemic varies across countries. Benmelech and Tzur-Ilan (2020) show that high-income countries, and those with high credit ratings, announced larger and non-conventional fiscal policy packages because the close to zero interest rates limited their policy options. Feyen et al. (2021) find that policymakers in richer and more populous countries reacted more quickly to the COVID-19 pandemic and introduced more policy measures in response to the pandemic. The authors also report that countries that belong to a monetary union intervened more frequently in their markets. Dinh (2022) suggests that the fiscal stimuli are, in particular, critical for developing countries as governments are expected to provide social protection for vulnerable populations to protect the labor supply.

This warrants an empirical investigation into whether the government’s economic support for households during the pandemic has been able to restore consumer confidence. As such, in this study, we investigate the association between the government’s household economic support and consumer confidence in OECD countries. More specifically, we examine which government economic support for households, income support policy, or debt/contract relief policy, is more appropriate to mend consumer confidence during the pandemic.

**Fig. 1:**
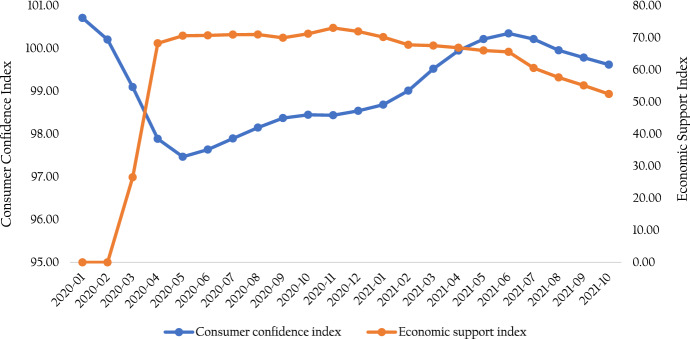
Evaluation of Consumer Confidence Index and Economic Support Index in OECD countries from January 2020 to October 2021. Source: https://www.bsg.ox.ac.uk/research/research-projects/covid-19-government-response-tracker & OECD (2021a, b, c), Consumer Confidence Index (CCI) (indicator). https://doi.org/10.1787/46434d78-en

Figure 1 illustrates the movements of the monthly consumer confidence and economic support indices from January 2020 to October 2021 in OECD countries. The indices moved in the same direction (although with some lags) since the beginning of the pandemic and it seems higher levels of economic support lead to a higher level of consumer confidence.

Utilizing the OxCGRT’s (2021) Economic Support Index (which includes households’ income support and debt/contract relief sub-indices) and OECD’s (2021a, b, c) Consumer Confidence Index and controlling for other important determinants of consumer confidence, the estimation results suggest that government economic support for households have a positive and significant impact on consumer confidence in the OECD sample economies. The impact is more apparent in emerging economies. We also find that the government’s income support policies have a stronger stimulating effect on consumer sentiment than debt/contract relief policies, which mainly benefit emerging countries. Generally, the results of this study lend empirical support to the government’s fiscal initiatives to recover an economy in a crisis.

Our contribution to the literature is twofold. First, while there have been some studies on the effect of fiscal and monetary policies on consumer confidence (e.g., Konstantinou and Tagkalakis 2011; De Boef and Kellstedt 2004), to the best of our knowledge, no empirical study has analyzed the macroeconomic policies-consumer confidence nexus during the COVID-19 pandemic, a crisis with significant health and economic costs. Second, no previous study has explicitly examined and compared the impact of the two household support policies (income vs. debt/contract relief) on consumer confidence across a large sample of emerging and advanced economies. Therefore, our results provide valuable new insights into the literature.

This article proceeds as follows: Sect. 2 describes the employed data, chosen variables, and estimation method; Sect. 3 presents the findings, and Sect. 4 concludes the paper.

## Data and methodology

### Sample

We use monthly data for 35 OECD countries, covering the period between January 2020 and October 2021. The sample countries are *Australia, Austria, Belgium, Brazil, Chile, China, Colombia, Czech Republic, Denmark, Estonia, Finland, France, Germany, Greece, Hungary, Indonesia, Ireland, Israel, Italy, Japan, Mexico, Netherlands, Poland, Portugal, Russia, Slovakia, Slovenia, South Africa, South Korea, Spain, Sweden, Switzerland, Turkey, United Kingdom, and the United States*. The choice of sample economies and period of study is dictated by data availability of the Consumer Confidence Index and Economic Support Index. According to the World Bank (2021), the sample accounts for more than 70% of the world’s GDP (constant 2015 US$) in 2020 which represents a large portion of the world’s economic activities.

In our estimations, besides the full sample analyses, we split the sample into two categories, depending on the level of economic development. The categorization of countries into advanced economies and emerging economies is based on the IMF definition. According to the IMF (2022), the following 11 countries in our sample are classified as emerging economies: Brazil, Chile, China, Colombia, Hungary, Indonesia, Mexico, Poland, Russia, South Africa, and Turkey. The rest of 24 countries are classified as advanced economies. The reason for running separate analyses for these sub-samples is to find whether the size and significance of the government’s economic support are different in advanced economies (vs. emerging economies). As can be seen in Table 5 in the “Appendix,” the fiscal support policies in response to the pandemic varied widely across countries, as advanced economies provided more significant support to their citizens and economies as a percentage of GDP.

### Dependent variable

The dependent variable of the study is consumer confidence. To measure this variable, we use the consumer confidence indicator obtained from the OECD (2021a, b, c). This indicator is the arithmetic average of the seasonally adjusted balances—that is, answers to questions about household finances. Such insights include households’ expectations about their savings, general economic situation, and national unemployment levels for the coming year. For example, the fourth question in the Consumer confidence indicator section of the Joint Harmonised EU Programme of Business and Consumer Surveys asks, “How do you expect the financial position of your household to change over the next 12 months?” The answers, which are a *5*-point Likert scale, ranging from “get a lot better” to “get a lot worse” in addition to an “I don’t know” answer. The average of the index over our sample period is highest in China (103.56) and lowest in Chile (95.75). Description of variables, data sources, and descriptive statistics are provided in Table 1. It is noteworthy that the OECD’s consumer confidence data have been widely used by researchers (e.g., Paradiso et al. 2014; Konstantinou and Tagkalakis 2011; Costola et al. 2022).

**Table 1:** Variable descriptions, data sources, and descriptive statistics in estimation sample

Variable	Description	Data source	Mean	Std. Dev
Consumer Confidence Index	“This consumer confidence indicator provides an indication of future developments of households’ consumption and saving, based upon answers regarding their expected financial situation, their sentiment about the general economic situation, unemployment, and capability of savings. An indicator above 100 signals a boost in the consumers’ confidence toward the future economic situation, as a consequence of which they are less prone to save, and more inclined to spend money on major purchases in the next 12 months. Values below 100 indicate a pessimistic attitude toward future developments in the economy, possibly resulting in a tendency to save more and consume less”	OECD (2021a)	99.09	2.09
Economic Support Index	“The index records measures such as income support and debt relief. It is calculated using all ordinal economic policy indicators” The ordinal economic policies are income support (for households) and debt/contract relief (for households)	OxCGRT (2021)	61.69	27.74
Income Support Index	E1_Income support (for households) Record if the government is providing direct cash payments to people who lose their jobs or cannot work Note: only includes payments to firms if explicitly linked to payroll/salaries Ordinal scale 0—no income support 1—government is replacing less than 50% of lost salary (or if a flat sum, it is less than 50% median salary) 2—government is replacing 50% or more of lost salary (or if a flat sum, it is greater than 50% of median salary) Blank—no data	OxCGRT (2021)	1.43	0.68
Debt/Contract Relief Index	E2_Debt/contract relief (for households) Record if the government is freezing financial obligations for households (e.g., stopping loan repayments, preventing services like water from stopping, or banning evictions) Ordinal scale 0—no debt/contract relief 1—narrow relief, specific to one kind of contract 2—broad debt/contract relief	OxCGRT (2021)	1.22	0.75
Composite leading indicator	“The composite leading indicator (CLI) is designed to provide early signals of turning points in business cycles showing fluctuation of the economic activity around its long-term potential level. CLIs show short-term economic movements in qualitative rather than quantitative terms”	OECD (2021b)	98.48	3.35
COVID-19 deaths	Monthly new confirmed COVID-19 deaths per million people	Our World In Data (2022)	74.47	106.41

### Variables of interest

The explanatory variable of interest is the government’s economic support in response to the COVID-19 pandemic. As a measure of this variable, we use the comprehensive Economic Support Index developed by the Oxford COVID-19 Government Response Tracker (OxCGRT 2021). The index measures all ordinal economic policy indicators. To gain a better understanding of the impacts of economic support policy on consumer confidence we conduct separate analyses on each sub-index of economic support policies for households: income support and debt/contract relief. Data for the economic support index are accessible daily. As our other variables are only available in monthly frequency, we convert the daily index into monthly series by taking the average of the daily values within each month, for each country.

### Control variables

Empirical studies have suggested a range of economic and political factors as determinants of consumer confidence including inflation rate, unemployment rate, economic activities (often measured by GDP or industrial production index), stock market index, interest rate, macroeconomic policies, media coverage of the economy, management of the economy, and political events (e.g., De Boef and Kellstedt 2004; Hollanders and Vliegenthart 2011; Ramalho et al. 2011; Paradiso et al. 2014; Konstantinou and Tagkalakis 2011).

Since there is no theoretical work that establishes the determinants of consumer confidence (Bachman and Sims 2012), most studies use ad hoc specifications based on their studies’ design and data availability. This has resulted in some contradictory findings. For instance, in a longitudinal study on OECD countries, Konstantinou and Tagkalakis (2011) show that the impacts of unemployment and inflation on consumer confidence are statistically insignificant. On the other hand, De Boef and Kellstedt (2004), using time-series regressions, find that inflation and unemployment have a significant and negative impact on consumer confidence in the USA.

Van Dalen et al. (2017) suggest an alternative way to capture the expectation about the future state of the economy by using OECD Composite Leading Indicators (CLI). CLI was developed in the 1970s to anticipate fluctuations in economic activities in the next six to nine months. This measure is developed using a range of forward-looking and country-specific indicators such as order books, confidence indicators, building permits, and long-term interest rates. For example, the Australian CLI is an aggregated measure of the following monthly economic indicators: (1) permits issued for dwellings, (2) order inflow figures, (3) production figures, (4) employment figures, (5) share prices, (6) terms of trade, and (7) 10-year government bond yield. Other studies have shown that similar indicators affect consumer confidence at both lagged and current levels (Soroka et al. 2015).

We follow Van Dalen et al. (2017) and include CLI, alongside the main variable of interest (economic support index), in our estimation model. In addition, given that the severity of fatality caused by the COVID-19 pandemic can hurt households’ confidence, we include monthly new confirmed COVID-19 deaths per million people. Data for this variable are collected from Our World In Data (2022). Finally, we control the local political events by including the country fixed effects in our estimations.

### Estimation method

We apply panel fixed effect to examine the relationship between government economic support and consumer confidence over time and across countries. The empirical model is specified as follows:

1 $$ {\text{CCI}}_{it} = \beta_{1} {\text{ESI}}_{it} + \beta_{2} X_{it} + \, \alpha_{i} + \mu_{t} + \, u_{it} $$

where CCI_*it*_ is the dependent variable (Consumer Confidence Index) where *i* is country and *t* is time (month); ESI_*it*_ represents Economic Support Index (and its two sub-components- Income Support Index and Debt/Contract Relief Index), *X*_*it*_ represents the control variables (composite leading indicator and monthly new confirmed COVID-19 deaths per million people), *α*_*i*_ (*i* = 1….*n*) is the unknown intercept for each entity (country fixed effects), *µ*_*t*_ is period fixed effects, *β*s are the coefficients and *u*_*it*_ is the error term. The country fixed effects account for all time-invariant differences across OECD economies, such as national cultural values which are not changing quickly. The period fixed effects control factors that vary across time but are constant across countries (Baltagi 2008). It is important to control for period effects given that all countries in our sample are affected by the global news about the pandemic development.

It should be noted that monthly new confirmed COVID-19 deaths per million and government economic support indicators are not strongly correlated. The correlation coefficients between *COVID New Deaths per Million* and *Economic Support Index*, *Income Support Index* and *Debt/Contract Relief Index* are 0.0793, 0.1132, and 0.0410, respectively. The low correlation between these variables is because some countries had high monthly new confirmed COVID-19 deaths per million but their governments did not support the households significantly (e.g., because of budget constraints). In contrast, some governments offered significant support to their households despite their low rate of COVID-19 death. Iran and Australia can be considered examples. Since the beginning of the pandemic, the Australian government provided several economic support policies for its households although the country did not experience a large number of COVID-related deaths during the early pandemic. The situation in Iran was very different. While the country had a high rate of COVID-19 deaths, the government could not support its households, mainly due to budget constraints and international economic sanctions.

## Findings

### Estimation results (with concurrent values of government economic support)

Panels A, B, and C of Table 2 report the estimation results when we include the Economic Support Index, Income Support Index, and Debt/Contract Relief Index as the key variables of interest, respectively. In all estimations, the Consumer Confidence Index is the dependent variable. Columns 1–3 of Table 2 show the results for the full sample, advanced economies, and emerging economies, respectively. The findings indicate that the Economic Support Index has the expected positive association with the Consumer Confidence Index and is statistically significant for the full sample, indicating that higher levels of government economic support for the household during the pandemic increase consumer confidence (see column 1 in Panel A of Table 2).

**Table 2:** Estimation results (with concurrent values of government economic support)

Dependent variable: Consumer Confidence Index			
	Full sample	Advanced economies	Emerging economies
	(1)	(2)	(3)
*Panel A: Aggregate Economic Support Index*			
Economic Support Index	0.008*** (3.62)	0.005* (1.86)	0.008** (2.32)
Composite Leading Indicators	0.066*** (2.86)	0.022 (0.69)	0.136*** (3.20)
COVID New Deaths per Million	− 0.004*** (− 10.08)	− 0.004*** (− 7.97)	− 0.003*** (− 3.54)
Country fixed effect	Yes	Yes	Yes
Period fixed effect	Yes	Yes	Yes
Within *R*-sq	0.62	0.68	0.53
Number of obs	697	482	215
Number of countries	35	24	11
*Panel B: Income Support Index*			
Income Support Index	0.299*** (3.24)	0.395*** (3.50)	0.100 (0.61)
Composite Leading Indicators	0.070*** (3.05)	0.022 (0.67)	0.151*** (3.54)
COVID New Deaths per Million	− 0.005*** (− 10.39)	− 0.004*** (− 8.27)	− 0.003*** (− 3.80)
Country fixed effect	Yes	Yes	Yes
Period fixed effect	Yes	Yes	Yes
Within *R*-sq	0.62	0.69	0.52
Number of obs	697	482	215
Number of countries	35	24	11
*Panel C: Debt/Contract Relief Index*			
Debt/Contract Relief Index	0.204*** (2.74)	0.009 (0.09)	0.413*** (3.16)
Composite Leading Indicators	0.064*** (2.73)	0.015 (0.47)	0.122*** (2.87)
COVID New Deaths per Million	− 0.004*** (− 9.96)	− 0.004*** (− 7.96)	− 0.003*** (− 3.29)
Country fixed effect	Yes	Yes	Yes
Period fixed effect	Yes	Yes	Yes
Within *R*-sq	0.62	0.68	0.54
Number of obs	697	482	215
Number of countries	35	24	11

*, **, ***Significance at the 10%, 5%, and 1% levels, respectively. *t*-statistics are in parentheses

These results are consistent with the Keynesian school of thought and several studies that argue expansionary fiscal policies lead to higher consumer confidence and so to higher demand and consumer spending (Alesina et al. 2015; De Boef and Kellstedt 2004; Konstantinou and Tagkalakis 2011).

The control variable (Composite Leading Indicators (CLI)) has the predicted sign and is statistically significant at a 1% level for the full sample, indicating there is a positive relationship between CLI and consumer confidence (see Panels A, B, and C of Table 2). This result is in line with the findings of Van Dalen et al. (2017). The results also show that number of new confirmed COVID-19 deaths per million people has a negative and significant association with consumer confidence across all specifications (see Panels A, B, and C of Table 2).

After analyzing the full sample data, we run the regressions for the sub-samples. As can be seen from panel A of Table 2, while the association between the Economic Support Index and Consumer Confidence Index is only significant at a 10% level for advanced economies, a stronger association exists between these variables for emerging economies. It means that households’ confidence in emerging economies is more sensitive to the government’s overall economic support during the pandemic than in advanced economies.

Next, we examine the relationship between two sub-components of the Economic Support Index (Income Support Index and Debt/Contract Relief Index) and consumer confidence in Panels B and C of Table 2. The coefficient of the Income Support Index is positive and statistically significant at a 1% level in the full sample (see the first column of Panel B in Table 2). The coefficient of the Debt/Contract Relief Index (*β* = 0.204) is smaller than the Income Support Index (*β* = 0.299) (the first column of Panel C in Table 2), meaning that income support is more effective than debt/contract relief to boost consumer confidence in the sample OECD countries. However, when we look at the subsample analyses, the findings of our panel fixed effect estimations suggest that this is the income support that can stimulate consumer confidence in advanced economies. In contrast, consumer confidence in emerging economies reacts better to the debt/contract relief economic support policies. The coefficient of income support is statistically significant for advanced economies (column 2 of Panel B) whereas the coefficient of debt/contract relief is significant and positive only for emerging economies (column 3 of Panel C).

The different impacts of economic support policies for households in emerging and advanced economies can be explained by their economic capacities to deploy public funds and resources to confront economic shocks (Kose et al. 2018). Developing countries have shallower financial markets and their debt is more subject to exchange rates and maturity risks. They often suffer from a lack of market-determined interest rates, low rates of interbank competition, and exchange rate intervention (Mishra et al. 2012). Moreover, developing countries’ less efficient tax systems and smaller tax bases make it more difficult to implement income support fiscal policies (Loayza and Pennings 2020). Ilzetzki et al. (2013) and Kraay (2014) provide evidence that the fiscal multiplier, as a measure of fiscal policy’s ability to stimulate economic output, is small in developing countries. As such, these factors may contribute to more popularity of debt/contract relief policies over the income support policies for households in emerging countries.

### Estimation results (with lag values of government economic support)

One may argue that government economic support does not have an immediate impact on consumer confidence but appears sometime later as the announcements of government economic policies have a delayed impact on households’ confidence. Therefore, we use the one-month lag of the Economic Support Index and its two sub-components in the panel fixed effects regressions. The results of these estimations are presented in Table 3. As can be seen in Panels A-C of Table 3, overall, the sign and significance of coefficients of variables of interest (government economic support indices) are almost similar to the concurrent values of these variables in Table 2.

**Table 3:** Estimation results (with lag values of government economic support indicators)

Dependent variable: Consumer Confidence Index			
	Full sample	Advanced economies	Emerging economies
	(1)	(2)	(3)
*Panel A: Aggregate Economic Support Index*			
Economic Support Index (− 1)	0.007*** (3.33)	0.003 (0.90)	0.011*** (2.80)
Composite Leading Indicators	0.067*** (2.91)	0.018 (0.56)	0.134*** (3.16)
COVID New Deaths per Million	− 0.004*** (9.80)	− 0.004*** (− 7.94)	− 0.003*** (− 3.21)
Country fixed effect	Yes	Yes	Yes
Period fixed effect	Yes	Yes	Yes
Within *R*-sq	0.62	0.68	0.54
Number of obs	696	481	214
Number of countries	35	24	11
*Panel B: Income Support Index*			
Income Support Index (− 1)	0.319*** (3.32)	0.450*** (3.73)	0.045 (0.26)
Composite Leading Indicators	0.069*** (3.00)	0.021 (0.64)	0.150*** (3.51)
COVID New Deaths per Million	− 0.004*** (− 10.23)	− 0.005*** (− 8.43)	− 0.003*** (− 3.71)
Country fixed effect	Yes	Yes	Yes
Period fixed effect	Yes	Yes	Yes
Within *R*-sq	0.62	0.69	0.52
Number of obs	696	481	214
Number of countries	35	24	11
*Panel C: Debt/Contract Relief Index*			
Debt/Contract Relief Index (− 1)	0.166** (2.20)	− 0.101 (− 1.07)	0.498*** (3.81)
Composite Leading Indicators	0.066*** (2.86)	0.012 (0.36)	0.126*** (3.03)
COVID New Deaths per Million	− 0.004*** (− 9.83)	− 0.004*** (− 8.02)	− 0.003*** (− 3.12)
Country fixed effect	Yes	Yes	Yes
Period fixed effect	Yes	Yes	Yes
Within *R*-sq	0.62	0.68	0.55
Number of obs	696	481	214
Number of countries	35	24	11

*, **, ***Significance at the 10%, 5%, and 1% levels, respectively. *t*-statistics are in parentheses

### Robustness checks with the GMM estimator

In addition to the panel fixed effects estimation method, we apply the system GMM estimator (Blundell and Bond 1998). To estimate the model, the system GMM estimator combines the first-differenced and levels equations, using both lagged levels and differences as instruments. To remove the individual country effect, system GMM is estimated using a first difference transformation. Moreover, system GMM retains the cross-country aspect of the data that is lost when we use the first-differenced GMM (Castelló-Climent 2008; Bjorvatn et al. 2012). In system GMM, we control for the high persistence of consumer confidence by including a year lag of it as an explanatory variable. We treat lags of the Consumer Confidence Index, Government Economic Support Index, and COVID New Deaths per Million as potentially endogenous variables and use 12 months lags of the instrumented variables as internal instruments.

Columns 1–3 of Table 4 show the estimation results using the Aggregate Economic Support Index, Income Support Index, and Debt/Contract Relief Index, respectively. We compute the Windmeijer correction for the standard errors for the two-step system GMM (Windmeijer 2005). The estimation results show that there is a positive and significant association between the government Aggregate Economic Support Index (mainly driven by the Income Support Index) and the Consumer Confidence Index. In general, these results reaffirm our earlier findings obtained from the fixed effects regressions. The diagnostic statistics are also satisfactory. The *Hansen* test does not reject the over-identification restriction, suggesting that the instruments are not correlated with the error term and are therefore valid for this analysis. The absence of first order serial correlation (AR(1)) is rejected while we accept the absence of second order serial correlation (AR(2)). Moreover, the one-year lagged dependent variable is positive and significant. The relatively high estimated coefficient of the lagged dependent variable shows its persistency, however, it is statistically different from unity in all models, indicating a stability of system GMM.

**Table 4:** System GMM estimation results

Dependent variable: Consumer Confidence Index			
	(1)	(2)	(3)
One-year lag of Consumer Confidence Index	0.757*** (2.80)	0.965** (2.29)	0.636** (2.42)
Aggregate Economic Support Index	0.032** (2.15)		
Income Support Index		0.983* (2.01)	
Debt/Contract Relief Index			0.771 (0.98)
Composite Leading Indicators	0.177 (0.50)	0.097 (0.54)	0.091 (0.24)
COVID New Deaths per Million	− 0.004 (− 0.89)	− 0.002 (− 0.81)	− 0.008 (− 1.24)
Observations	343	343	343
Number of instruments	47	46	47
Hansen test of overid. Restrictions (*p* value)	0.947	0.939	0.951
Arellano-Bond test for AR(1) in first differences (*p* value)	0.005	0.012	0.0008
Arellano-Bond test for AR(2) in first differences (*p* value)	0.552	0.136	0.763

*, **, ***Significance at the 10%, 5% and 1% levels, respectively. *t*-statistics in parentheses are based on Windmeijer correction for the standard errors in two-step system GMM estimation. Time dummies are included (not shown)

We also check whether including additional control variables such as unemployment rate, inflation rate, retail trade, and industrial production, would impact the relationship between governments’ economic support for households during the pandemic and consumer confidence (see the estimation results in Table 6 in “Appendix”). Including additional control variables does not change the significant and positive effect of government (income) support on consumer confidence. The new control variables included do not show a statistically significant association with consumer confidence. The negative and highly significant effect of COVID-19 fatality on consumer confidence remains the same. It should be noted that the number of sample countries declines from 35 to 26 in this analysis due to missing values for the additional control variables. Data for Unemployment Rate (% of labor force), Inflation Rate (growth in Consumer Price Index), Industrial Production Index are obtained from OECD (2022). Data for Total Retail Trade (Volume) Seasonally Adjusted Index are collected from OECD.Stat (2022).

## Conclusion

Since the beginning of the pandemic, most governments around the world have implemented several economic policies to minimize the adverse impact of a health crisis on economic activities. It is believed that economic support can boost consumers’ and firms’ economic confidence which leads to increases in consumption and investment expenditures. In this study, we use monthly data from January 2020 to October 2021 from 35 OECD countries to test if the government’s economic support for households has stimulated consumer confidence. Our regression results suggest that economic support is indeed effective in positively enhancing households’ perception of the overall macroeconomic condition. Further analyses show that the government’s pandemic income support for households has a stronger impact than debt/contract relief on consumer confidence in advanced economies. However, we find that debt/contract relief is a more effective policy to boost confidence in emerging economies.

Our study contributes to the long-standing literature on economic policies-consumer confidence nexus by testing this important link during the worst economic crisis after World War II. Our findings also have implications for policymakers in the OECD countries. First, economic support for households should be available during the crisis because it has a favorable impact on households’ confidence which is one of the main determinants of households’ willingness to spend on goods and services (especially durable goods). Such support needs to be provided to sustain the livelihood of households in the short run even if it may cause a higher inflation rate in the long run. Second, although both income support and debt relief supports are important for consumer confidence, policymakers need to pay more attention to income support in advanced economies and debt/contract relief in emerging economies.

In this study, we only use the OECD countries’ sample due to the availability of consumer confidence data for these countries. Future studies may repeat our research for other countries if the data become accessible from early 2020 onwards.

## Appendix

See Tables 5 and 6.

**Table 5:** Economic support in response to the COVID-19 Pandemic (% of GDP)

Country	Additional spending and forgone revenue (% of GDP)	Equity, loans, and guarantees (% of GDP)
Australia	18.37	1.8
Austria	15.198	2.82
Belgium	8.18	11.88
Brazil	9.24	6.15
Chile	12.68	2.50
China	4.78	1.30
Colombia	4.62	5.38
Czech Republic	9.2	15.5
Denmark	3.41	15.63
Estonia	5.80	5.088
Finland	4.78	7.37
France	9.6	15.2
Germany	15.3	27.8
Greece	17.5	3.688
Hungary	11.5	4.2
Indonesia	9.33	0.88
Ireland	11.548	2.681
Israel	10.3	3.7
Italy	10.9	35.3
Japan	16.7	28.3
Mexico	0.65	1.20
Netherlands	10.3	4.3
Poland	6.46	4.82
Portugal	6.0	5.7
Russia	5.01	1.46
Slovakia	5.9	1.7
Slovenia	9.4	6.6
South Africa	5.28	4.08
South Korea	6.40	10.13
Spain	8.4	14.4
Sweden	4.15	5.25
Switzerland	7.91	6.22
Turkey	3.51	9.63
United Kingdom	19.3	16.7
United States	25.5	2.4

*Sources*: Database of Country Fiscal Measures in Response to the COVID-19 Pandemic; and IMF staff estimates

Percentages of GDP are based on October 2021 World Economic Outlook. Country group averages are weighted by GDP in US dollars adjusted by purchasing power parity

**Table 6:** Estimation results (with additional control variables)

Dependent variable: Consumer Confidence Index			
	(1)	(2)	(3)
Aggregate Economic Support Index	0.005 (0.91)		
Income Support Index		0.347** (2.14)	
Debt/Contract Relief Index			0.078 (0.35)
Composite Leading Indicators	0.028 (0.45)	0.031 (0.54)	0.026 (0.41)
COVID New Deaths per Million	− 0.004*** (− 5.18)	− 0.004*** (− 5.04)	− 0.004*** (− 5.20)
Unemployment Rate	0.143 (1.45)	0.141 (1.42)	0.143 (1.46)
Inflation Rate	− 0.029 (− 0.21)	− 0.033 (− 0.25)	− 0.035 (− 0.25)
Retail Trade Index	− 0.002 (− 0.11)	− 0.004 (− 0.21)	− 0.002 (− 0.11)
Industrial Production Index	− 0.014 (− 0.76)	− 0.014 (− 0.82)	− 0.014 (− 0.77)
Country fixed effect	Yes	Yes	Yes
Period fixed effect	Yes	Yes	Yes
Within *R*-sq	0.65	0.66	0.65
Observations	492	492	492
Countries	26	26	26

*, **, ***Significance at the 10%, 5%, and 1% levels, respectively. Robust *t*-statistics are in parentheses
